# A Novel Low-Cost Sensor Prototype for Monitoring Temperature during Wine Fermentation in Tanks

**DOI:** 10.3390/s130302848

**Published:** 2013-02-28

**Authors:** Beatriz Sainz, Jonathan Antolín, Miguel López-Coronado, Carlos de Castro

**Affiliations:** 1 ETSI de Telecomunicación, Universidad de Valladolid, Campus Miguel Delibes, Paseo Belén 15, 47011 Valladolid, Spain; E-Mails: jantlaz@gmail.com (J.A.); miglop@tel.uva.es (M.L.-C.); 2 EATCO Research Group, Department of Computer Science, University of Cordoba, Campus de Rabanales, 14071 Córdoba, Spain; E-Mail: ma1caloc@uco.es

**Keywords:** wine fermentation process, biosensor, temperature, Bluetooth, low cost, usability

## Abstract

This paper presents a multipurpose and low cost sensor for temperature control over the wine fermentation process, in order to steadily communicate data through wireless modules in real time to a viticulturist's mobile or fixed device. The advantage of our prototype is due to the fact that it will be used by small winemakers in the “Ribera del Duero” area, and as it is a cheaper sensor and easy to use for the control and monitoring of the grape fermentation process, it will probably be used by other business men with the same necessities in the region. The microcontroller MSP430G2553 is among the components that make up the sensor, that are integrated onto a motherboard. It communicates with the RN-42 Bluetooth module through an UART interface. After verifying that all elements are working correctly, the parts are assembled to form the final prototype. This device has been tested in a winery in the region, fulfilling the initial project specifications.

## 1. Introduction

The wine fermentation process is exothermic, that is, it frees energy as heat, increasing the must's temperature. The microorganisms that process the grapes' sugar need a certain temperature (about 30 degrees Celsius) in order to transform must into wine. If these microorganisms are exposed to higher temperatures than 55 degrees Celsius during five minutes or more they die and wine is not processed. That is the reason why an exhaustive control of the temperature is needed during all processes.

As Wang *et al.* reveal in their work [1], in some cases high temperature sensors capable of operating in harsh environments are necessary disaster prevention from structural or system functional failures due to increasing temperatures and building upon the limitations of most of the existing temperature sensors. In this study a commercial transducer is applied to the monitoring task because we never reach higher temperatures.

Previous researchers have developed mechanisms to determine the rheological properties of fluids [2] and to evaluate the antioxidant capacity of substances [3]. Microbial biosensors have been used for sensing phenolic compounds in commercial wines [4], or different methods have been applied to the measurement of sulfite in wines [5]. Systems have also been developed, based on the use of e-noses and e-tongues, to characterize and classify wines according to grape variety and geographic origin [6–10]. Using a monitoring system improves the quality of wines. For a viticulturist the control during winemaking of the must fermentation process is as important as measuring a number of grape quality parameters, including soluble solid content, reducing-sugar content, titratable acidity, pH-value, tartaric acid and malic acid contents and sensory attributes [11], for which several techniques have been developed for the assessment of chemical changes in the main internal quality properties of wine grapes during on-vine ripening and at harvest [12–14].

It was considered that in industrial applications, agricultural areas, or any other field the use of wireless links reduces the installation cost in comparison to wired systems, especially when the area to be covered is large. The locations at which the sensors are placed are usually harsh environments. When wireless sensors are considered, either a single device or a Wireless Sensor Network-WSN [15–19] can be used, and there are some important issues to be considered such as autonomy, transmission frequency band, interferences, obstacles, cost, *etc.* For this project different solutions were analysed: Bluetooth, WiFi and ZigBee [20]. The WiFi technology was discarded because the sensor did not need a high transmission rate, and the cost and processing capacity were really high compared to the other two technologies.

If we compare the features of Bluetooth [21] and ZigBee [20], even though both have a similar network range, the rates of transmission offered by Bluetooth are higher than those of ZigBee. Furthermore, as Bluetooth is more popular than ZigBee, there are a lot of devices for this wireless technology available on the market, thus it is possible to develop applications for mobile phones, PDAs, *etc.* for the receiver data client, and in a way not to be restricted to only computers. Bluetooth turns out to be the most appropriate technology for its low implementation cost, its higher transmission rate, low power, and high market penetration rate.

As a result of this project, after analysing the microcontrollers and best suited sensors to the needs, a multipurpose very low cost sensor that sends data through a Bluetooth module was developed. It provides the viticulturist with a full and exhaustive control of the wine's temperature through an easy application installed on a mobile device. This application is very simple to use and displays the data collected by the prototype. Throughout all the development, the main objective was to reduce the cost of the final prototype so as to compete with other sensors available on the market which, compared to our prototype, have the drawback of being a high cost solution developed by private enterprises.

## 2. The Portable Prototype: Parts and Connections

### 2.1. Description of the MSP430 Launch Pad Used in this Work

The microcontroller is the heart of the system. It is responsible for processing information obtained by the transducers, turning this information from analog into digital and sending it to the destination, in our case, via a Bluetooth module. In order to select this component we considered the size, because we sought to develop a small portable device which is to be located inside the wine barrels. Other considerations that made us choose this device were its high computing power and its ultra-low consumption which determined the system's autonomy. This sensor is especially developed for small wine cooperatives. As in these places members don't want to invest too much in technology, we therefore, looked for the cheapest components that were easy to integrate and programme to thereby achieve our objectives.

**Figure 1.**
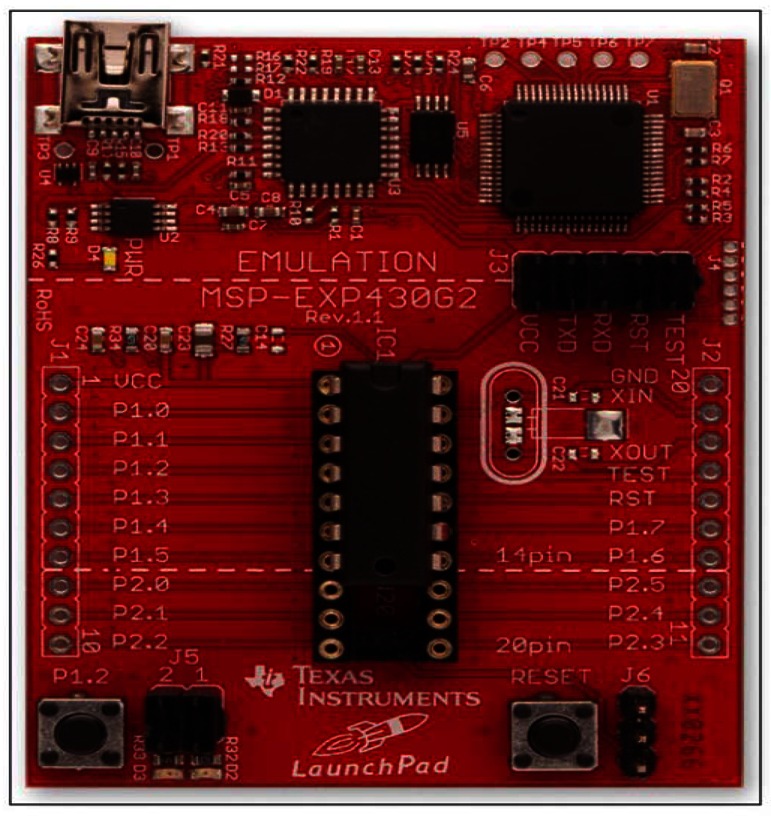
MSP430 Launch Pad [22].

Among all manufacturers that offered commercial solutions, we chose the MSP430 family developed by Texas Instruments [22]. This company offered an easy and economical development board, free software, low cost microcontroller, low power consumption and a full help in their wiki. These reasons made this family the best option to develop our prototype. MSP430 Launch Pad (Figure 1) includes a communication module port with the computer, debug mode for testing our programmes, access to input/output microcontroller's ports, Universal Asynchronous Receiver/Transmitter Interface (UART) port, and the necessary software and drivers for the computer [22].

### 2.2. Description of the MSP430G2553 Microcontroller

The choice of the MSP430G2553 [23], from among all microcontrollers developed by Texas Instruments, was due to its compatibility with the MSP430 Launch Pad, being included in the development kit, incorporating an internal temperature sensor and an integrated Analog-to-Digital Converter (ADC), being programmable in C, its low price and low power consumption. Its main features are described in the Texas Instruments' user guide. Table 1 shows the pins used for the prototype development.

#### 2.2.1. Description of the Integrated ADC and Temperature Sensor

The ADC converter module supports fast, 10 bit analog-to-digital conversions. The ADC10 core is configured by two control registers, ADC10 control register 0 (ADC10CTL0) and ADC10 control register 1 (ADC10CTL1). The core is enabled with the ADC10ON bit (bit 4). Each time the ADC module prepares and stores a conversion result in the ADC10MEM, the Data Transfer Controller (DTC) is triggered without any software intervention. The Central Process Unit (CPU) is halted to avoid any data bus connection during the DTC transfer. When the DTC operation is finished, the CPU goes on with the job [23]. To activate the integrated temperature sensor in the microcontroller we must choose the analogical input for this sensor. When this sensor is used, the microcontroller uses the internal voltage reference. The operating mode is the same as when an external channel for the conversion is used. The way to access to the ADC registers is the same.

#### 2.2.2. Universal Asynchronous Receiver/Transmitter Interface (UART)

By means of this interface we connect the MSP430G2553 to the RN-42 Bluetooth module. UART is a standard communication port. Its main function is to convert the information measurement by the sensor from parallel to serial in order to send it to Bluetooth module, and *vice versa*, from serial to parallel from the Bluetooth module to the MSP430G2553. In our prototype we only needed to connect the UART port of both devices. For that, we connected the microcontroller's output transmitter pin to the Bluetooth module's input receiver, and conversely, the Bluetooth module's transmitter output to the microcontroller's receiver input. We didn't need the rest of the UART pins.

### 2.3. Description of the Bluetooth Roving Networks RN-42-SM with UART Interface

**Figure 2.**
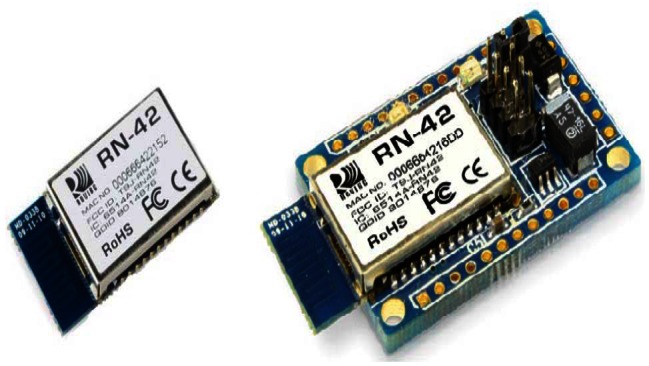
The RN-42 Roving Network Bluetooth module is on the left. On the right it is the RN-42 Roving Network Bluetooth module integrated in the RN-42-SM board [25].

To choose the Bluetooth module we analysed different solutions from diverse leading manufacturers. The most suitable was selected taking into consideration the small size, low price, low power consumption and an easy configuration. Among all possible solutions, we found the WT-12A from Bluegiga [24] and the RN-42 [25] from Roving Networks, with similar features. The reasons why we chose the RN-42 were because it was cheaper than the other and it could be configured for wireless use. We certainly worked with the RN-42-SM module based on RN-42 Roving Network Bluetooth chip [25]. Figure 2 shows both modules.

Some of its features are [25]:

- UART interface.
- UART rate transmission between 1,200 bps and 3 Mbps.
- Work voltage between 3.3 V to 5 V.
- Low power consumption technology. From 8 mA to 30 mA.
- Bluetooth profiles included: Serial Port Profile (SPP), Radio Frequency COMMunication (RFCOMM), Host Controller Interface (HID) and etcetera.

**Figure 3.**
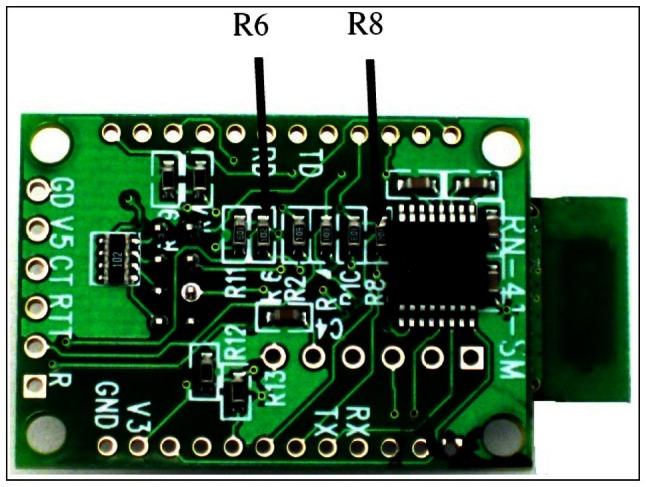
Resistances eliminated [25].

It is supplied with UART interface, like our MSP430G2553 microcontroller, that we used to make the connection between both devices. In order to configure our Bluetooth module we needed to know the features to implement. We had to activate the UART interface to transmit data and to define the SPP profile that was specifically developed to eliminate cabling. In our development we used the Header B. To use these pins the R6 and R8 resistances must be removed (as shown in Figure 3) cancelling the RS-232 chip.

Table 2 indicates the connections made so as everything works properly. Criterion: if the signal gets out from Bluetooth module, it is out, and if the signal gets in, it is in.

## 3. Development of the Prototype

### 3.1. Using the C Programme

**Figure 4.**
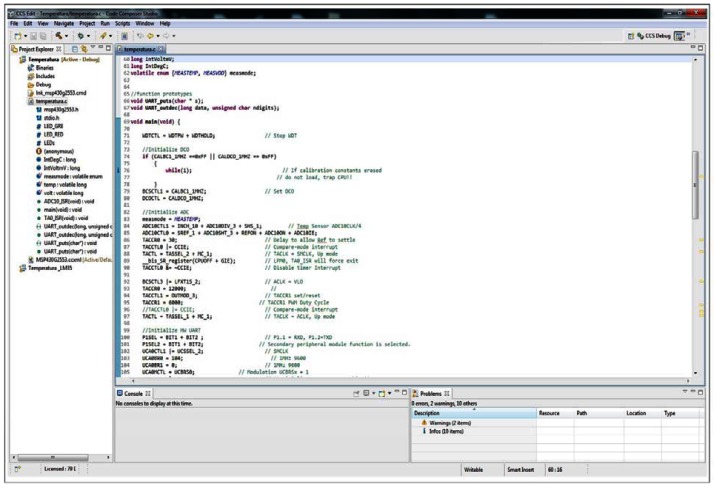
Code Composer Studio v5 (CCSv5).

To launch our prototype, we used especially designed software programmed by Texas Instruments (Figure 4), which allowed us to perform the debugging process through the development kit after installing the drivers in our computer [26]. The operation of this environment was really easy. The programme developed in C language was embedded in the microcontroller. We compiled our project with this software and executed the debugger to load the program into the microcontroller. Later, the microcontroller without PC could be used.

### 3.2. Development of the Design of Circuit

**Figure 5.**
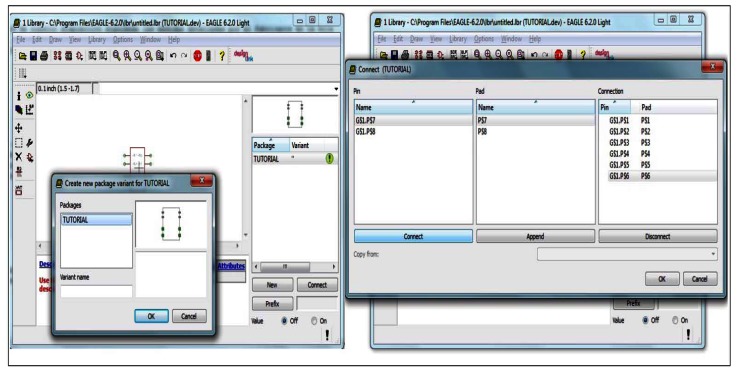
Bluetooth module library development.

Once tested over the launch pad that everything was working properly, we proceeded with the assembly. Through the design tool called Easily Applicable Graphical Layout Editor (EAGLE), v6, a product of Cadsoft Computers [27], we made a connection scheme in order to realize the connection over the test bed. Figure 5 shows some steps that we made to develop the encapsulated in the EAGLE since some component layouts were not included in the component library and the connection of the pins.

**Figure 6.**
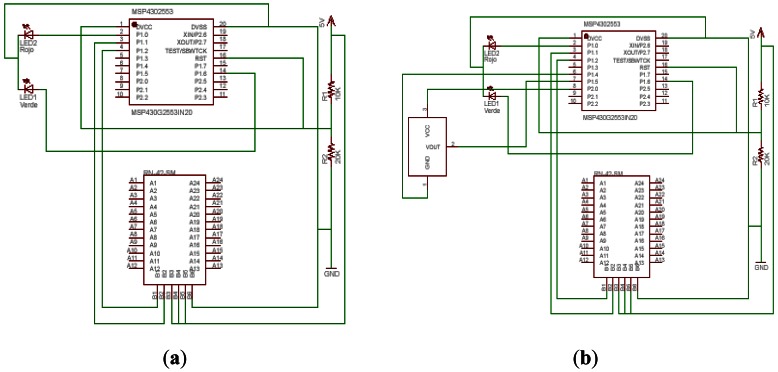
(**a**) Internal temperature sensor schematic design is on the left. (**b**) On the right, LM35DZ external temperature schematic sensor.

Figures 6 show the schematic designs of the connections. First using the internal sensor, and then using an external temperature transducer [28].

**Figure 7.**
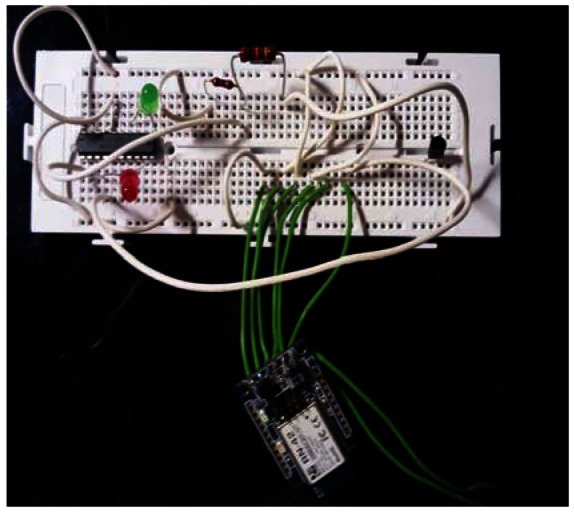
Assembly of the components on the test bed.

From these schemes we assembled the prototype (Figure 7). The parts used and the final costs are in Table 3.

The price will vary depending on the number of devices bought, discounts negotiated with vendors of the more important components on the board and minimum order quantities. We estimate that the approximate cost of this prototype development, including the Printed Circuit Board (PCB) manufacture, the board itself and other components like RN-42 Bluetooth module, is 49,50 € per unit (minimum order values apply for custom designed products of 100 units).

## 4. Results and Discussion

**Figure 8.**
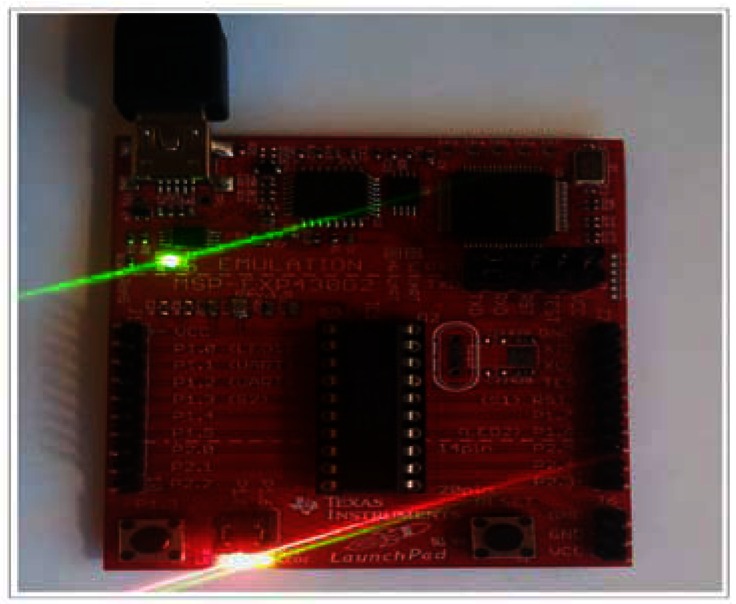
LED blinking.

After installing the control software into the microcontroller, thanks to the development board of Texas Instrument, we verified if it was recording the correct temperature values as it was programmed in the code (see Figure 8).

**Figure 9.**
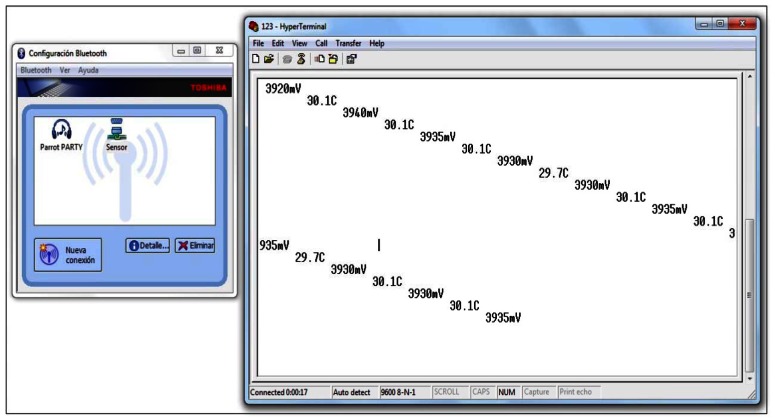
Receiving temperature data via Bluetooth.

The next step was to check the communication with the microcontroller through the COM port. To know what the assigned COM port was, we could consult the “Device Manager” in the Windows^®^ “Control Panel”. The number was assigned automatically by Windows^®^ and it could vary each time in function of the rest of the free ports. In the next step we made a connection with the microcontroller through “Hyperterminal”. Once checked that wired data could be received, we verified the wireless connection and equipped the Bluetooth module with a long life battery in order to connect and configure it through “Hyperterminal” (Figure 9). The battery used was a 4.5 V 3LR12. This power supply was sufficient during all process of data collection: two weeks. Even though during the development we used a PC, we tested our prototype over different platforms (laptop, PDA, Android, *etc.*), with integrated Bluetooth, and more portability and usability were obtained. All that was needed was to pair the prototype with the mobile device and in the same way we obtained a COM port. We opened again the terminal programme and configured the connection. Everything was ready to work without wires. Although the delay in sending data was not a significant parameter for this study, we analysed it in the laboratory and it was observed that it could be ignored. We connected the Bluetooth module UART port with the microcontroller UART port and opened the terminal program used in the last Bluetooth connection. Then we had to select 9,600 in the bits per second field.

**Figure 10.**
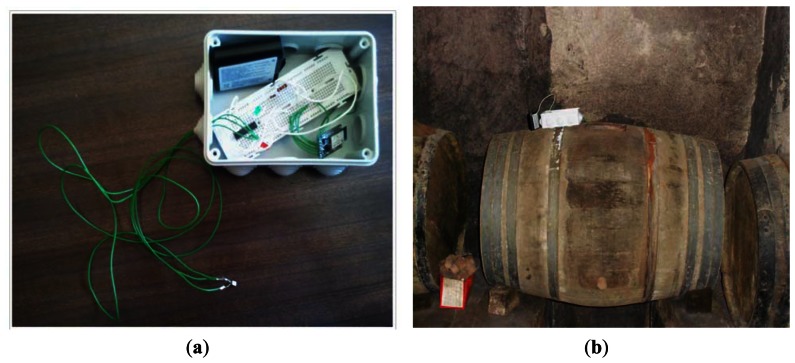
(**a**) Prototype for temperature measurement. (**b**) Data collection by novel wireless prototype in winery cellar of Dueñas, Palencia, Spain.

Figure 10(a) shows the circuit inside the box and outside the temperature transducer. It is protected by a resistant plastic material to guarantee the impermeability inside a wine barrel, avoiding a short circuit, because it is introduced like a catheter to measure the temperature in real time. As illustrated in Figure 10(b), the novel wireless prototype was assessed in a wine cellar in the Dueñas region of Palencia, Spain. The data collection process was performed for 15 days. It was time to ferment the wine and we have not been able to collect data over long periods of monitoring.

**Figure 11.**
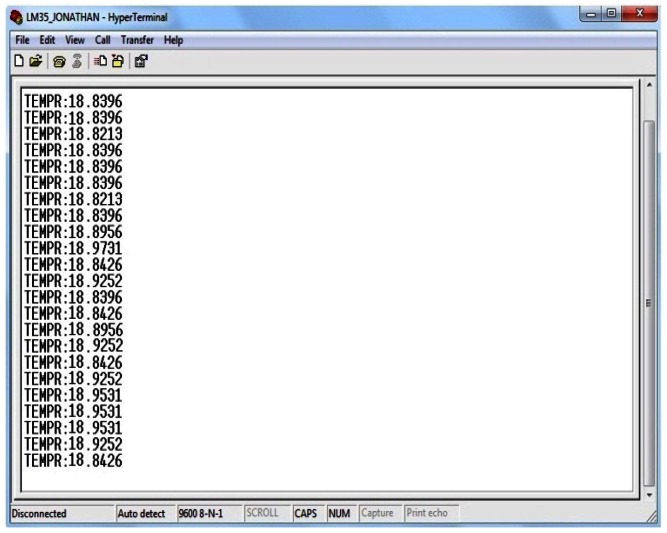
Data detection with LM35DZ.

Likewise the result with the integrated temperature sensor was validated; the measurements given by the LM35DZ transducer were checked (Figure 11).

**Figure 12.**
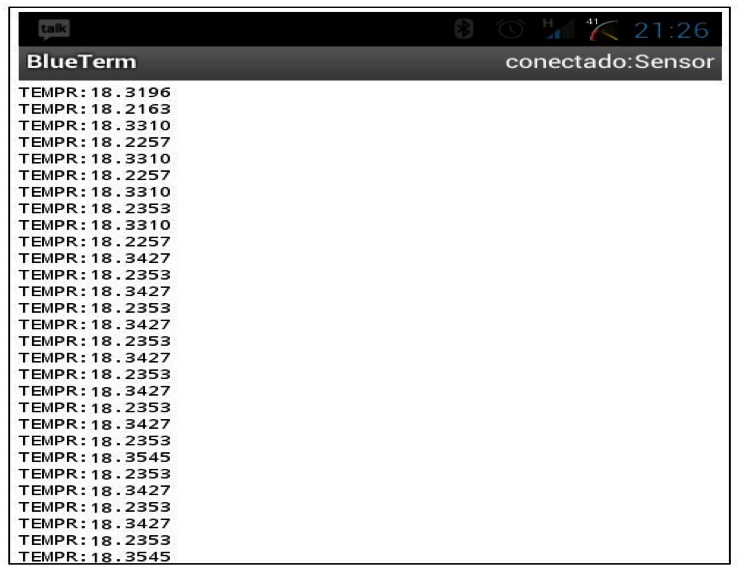
Receiving data via a terminal using the Android system.

Once the hardware side was validated, we focused our efforts on the received data control. The best way to control the measured value is through a terminal programme because commands can be sent to the device quickly. This kind of client consumes very few device resources where it is executed and its interface is simple. Nevertheless, it is also possible to develop visual programmes which is responsible for taking and processing data automatically. The obtained results are displayed in Figure 12. On the screen, we can read the data received from the sensor on an Android mobile phone, a Linux-based operating system for mobile devices.

Currently we have not found any low cost thermometer for measuring liquids effective enough to communicate data through a wireless module steadily to the viticulturist's device. We compared our prototype with a precision commercial thermometer, the HI 98509-01 [29]. This is an accurate temperature tester from Hanna Instruments measuring from −20 to 100 degrees Celsius. The stainless steel probe can be dipped into any tank to measure the temperature. The disadvantage is the high price, around 90.75 €. The cost of our prototype is less than 50 €. Another disadvantage of the HI 98509-01 is the fact that it cannot communicate data through a wireless module in real time.

Temperature changes that occurred during the short period of time in which the data was taken, corresponded to normal values. The temperature range measured was from 14 to 28 degrees Celsius. Identical values were obtained with the HI 98509-01. In the winery trained personnel set out to test the difference between temperatures taken with HI 98509-01 *vs.* our prototype. The aim was to verify whether our prototype could be a plausible option for measuring temperature for estimation. The temperature with HI 98509-01 was taken for a total of 2 hours every day for two weeks. This is the time of the red wine fermentation process.

**Figure 13.**
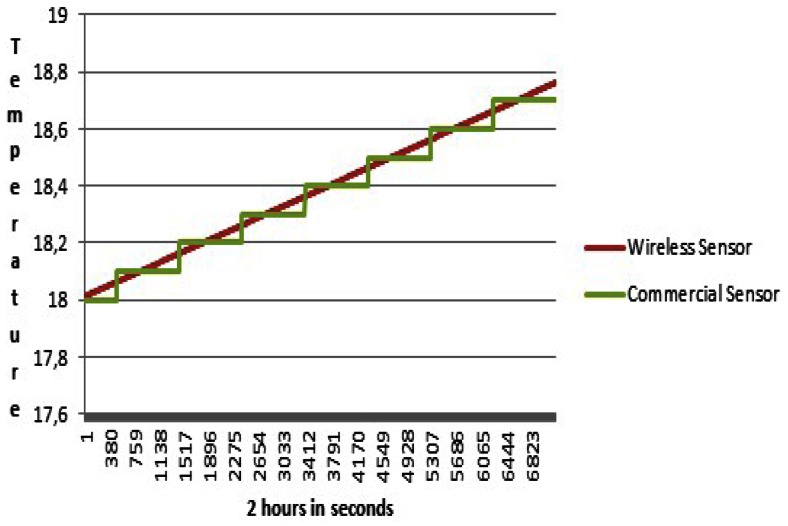
Comparison of temperature measurement devices for 2 hours on the 2nd day.

**Figure 14.**
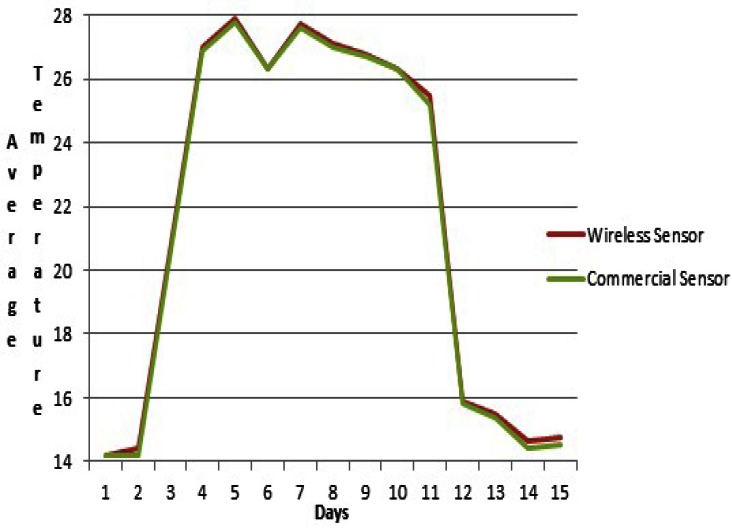
Average temperature value register: HI 98509-01 *vs.* prototype.

We can observe in Figure 13 the comparison of temperature measurement devices for 2 hours on the second day. No differences within the two technical methods can be observed. The margin of error is negligible and demonstrates the validity of the data. That is clearly shown in Figure 14. The values of rthe ed line are transmitted in real time and steadily to the viticulturist's device. The green line shows the temperature of the commercial HI 98509-01thermometer. These values were taken at regular intervals of time.

## 5. Conclusions

In this study, we have analysed a wide variety of microcontrollers that fulfilled the specifications of our project. We finally decided that the best was the MSP430G2553. The sensor choice was conditioned by the relation between quality and price of the system. Although there are a lot of sensors on the market which could measure temperature and humidity, the selection of this particular sensor was due to its accuracy when measuring, its small size and the way it sends information. Likewise, it was decided that the wireless technology that best suited the purpose of the project was the Bluetooth technology.

In this project a prototype was designed using the MSP430G2553 microcontroller. The ADC converter from the microcontroller was connected to the LM35DZ temperature transducer. This device was protected from the existing dampness in a wine cellar through an airtight electrical box. Through a hole applied in the box a wire with the transducer was pulled out from inside. After the validation of the data in the laboratory, during the testing process, the hole was sealed with silicone to ensure the integrity of the box. The catheter was submerged and was kept in contact with the must during all the fermentation process. When the Bluetooth link was not active, the radio of the RN-42 Bluetooth module was automatically turned off. It remained off during about the 85% of the time, promoting energy savings. The temperature values are transmitted in real time and steadily to the device. All transmitted data are processed in the user terminal. Through a terminal program we control the measured value. This client consumes very few device resources and its interface is simple. The study shows our prototype can be considered as a possible alternative to fixed-temperature detectors for measuring temperature of the fermentation process inside a tank. The Bluetooth module linked to the terminal device will permit the viticulturist exhaustive control of the final temperature through an easy application which permits him to study the data collected by our prototype and send it via wireless at a low cost. The portability, the lower price and the autonomy of our prototype were key features in our development project.

## Figures and Tables

**Table 1.** Pinout of MSP430G2553.

**PIN**	**TYPO**	**DESTINATION**
1	In	VDD. 5V.
2	Out	Red LED
3	In	Tx Bluetooth module
4	Out	Rx Bluetooth module
14	Out	Green LED
16	In	5V (Reset, Active low)
20	In	GND
Reset	Not in use	Not in Use

**Table 2.** Connecting pins on the RN-42-SM.

**PIN**	**TYPO**	**DESTINATION**
1	In	UART MSP430G2553 Transmitter Pin 4 MSP430G2553
2	Out	UART MSP430G2553 Receiver Pin 3 MSP430G2553
3	Out	5 V
4	In	5 V
6	VDD	5 V
6	GND	Ground

**Table 3.** Parts used and total cost of the prototype.

**COMPONENTS**	**PRIZE (€)**
Resistance 10k	0.18
Resistance 20k	0.18
Green LED	0.15
Red LED	0.18
Test Bed	10.35
MSP430G2553	1.91
RN-42-SM	32.18
External electrical junction box (160 × 115 × 70 mm)	3.7
TOTAL	48.83
